# Vitamin D3 and erythropoietin protect against renal ischemia-reperfusion injury via heat shock protein 70 and microRNA-21 expression

**DOI:** 10.1038/s41598-020-78045-3

**Published:** 2020-12-01

**Authors:** Mohammad Ghasem Golmohammadi, Shokofeh Banaei, Kazem Nejati, Mir Mehdi Chinifroush-Asl

**Affiliations:** 1 Department of Anatomy, School of Medicine, Ardabil University of Medical Sciences, Ardabil, Iran; 2 Department of Physiology, School of Medicine, Ardabil University of Medical Sciences, Ardabil, Iran; 3 Pharmaceutical Sciences Research Center, Ardabil University of Medical Sciences, Ardabil, Iran; 4 Department of Pathology, School of Medicine, Ardabil University of Medical Sciences, Ardabil, Iran

**Keywords:** Physiology, Nephrology

## Abstract

Kidney ischemia reperfusion (IR) contributes to the development of acute kidney injury. The hypoxic conditions in ischemic damage lead to oxidative stress and apoptotic cell death. We investigated the effects of vitamin D3 (Vit D) and erythropoietin (EPO) on microRNA-21(miR-21) expression in renal IR. Wistar rats were divided into five groups including the control, vehicle + IR, Vit D + IR, EPO + IR, and Vit D + EPO + IR groups. The animals were unilaterally nephrectomized and subjected to 45 min of renal pedicle occlusion followed by 24 h reperfusion. Vitamin D3 and EPO were administered prior to ischemia. After 24 h reperfusion, the kidney samples were collected for the detection of miR-21, heat shock protein 70 (hsp70) and caspase-3 expression levels. Kidney IR significantly increased the expression of miR-21, hsp70 and capase-3 and blood urea nitrogen (BUN)-Cr levels. Treatment with vitamin D3 and EPO significantly decreased the BUN-Cr levels and hsp70 and caspase-3 expression. Also, the co-administration of two drugs significantly increased miR-21 expression. It seems that vitamin D3 or EPO administration could protect the kidney against IR injury. However, vitamin D3 and EPO co-treatment was the most effective compared with the other treatment groups.

## Introduction

Kidney ischemia-reperfusion injury (IRI), the important cause of acute kidney injury (AKI), is a major complication after renal transplantation in clinical setting. Ischemia-reperfusion (IR) injury is a main contributor to leading cause of death worldwide. The hypoxic state arisen due to ischemic injury leaves tissues more sensitive to oxygen once perfusion is restored. However, reperfusion contributes to cellular damage and reactive oxygen species (ROS) production. Moreover, the initial hypoxic condition and reperfusion leads to activation of immune and inflammatory responses which can result in necrosis or apoptosis by damaging cellular DNA, proteins and cellular integrity. Therefore, it is essential to develop interventions to prevent ischemia-induced AKI^1,2^.

Micro RNAs (miRNAs) are short, endogenous, single stranded noncoding RNAs which contain approximately 22–25 nucleotides. They modulate gene expression by hybridization to messenger RNA (mRNA) leading to translational degradation of the target mRNA^3^. As a hematopoietic miRNA, miR-21 can be induced by hypoxia conditions such as renal IR injury and upregulated in kidney diseases and it might be used as a diagnostic biomarker in renal diseases. Previous studies have identified the induction of miR-21 through cell intrinsic as well as extrinsic pathways is vital in preventing tubular epithelial cell (TEC) apoptosis and an essential role is assumed for TGF-β (tubular growth factor-β) in renal IRI. Furthermore, it has been suggested that the induction of miR-21 expression in TEC occurs as the result of TGF-β signaling. Also, in the kidney, fibrosis is probably mediated by TGF-β signaling, as well as hypoxia or ischemia, through their effect on miR-21 expression. In TEC, TGF-β stimulation and IR lead to increased processing of pri-miR-21 by which TGF-β affects miR-21 expression. In renal undergoing IRI, pri-miR-21 is generally upregulated at early time-points, although miR-21 is usually upregulated at all time-points. The expression of miR-21 is gradually elevated following IR damage. All these findings imply an important role for miR-21 during renal IR injury^4^.

MiR-21, an anti-apoptotic miRNA which plays a role in several processes that occur as a result of IRI. Induction of hypoxia or ischemia in tubular epithelial cells results in upregulation of miR-21. As a result, the overexpression of miR-21 leads to reduced cell apoptosis. Also, miR-21 is found to exhibit a protective role against hypoxia-induced cell apoptosis and inflammation in the IR injury by inhibiting the caspase 3-induced apoptosis. Therefore, miR-21 may play a major role in the responses to ischemic injury and attenuate cell damage via regulating the programmed cell death and Akt pathway^5^. The pro-apoptotic protein (caspase-3) is an important apoptosis-related protein within the intrinsic apoptotic pathway. Caspase-3 is well known to act downstream of Bcl: Bax, and plays a major role in the execution of apoptosis. The activity of caspase-3 has been considered as an index of the process of apoptosis^6^.

Heat shock proteins (HSPs) are abundant cellular proteins that play important roles in mediating protein assembly, folding or refolding stress, denatured proteins and initiating cell death. The expression of HSPs can be upregulated by various stressors in a process termed heat shock response. Their clinical application such as ischemic conditions in transplantation and major surgeries is due to the cytoprotective effects of hsps. Hsp70 within the cells is located in the cytosol, mitochondria and nucleus. Hsp70 is induced by stressed cells to counter cellular damage and hasten recovery. Hsp70 induction ameliorates renal tubule-interstitial fibrosis and renal tubular epithelial cell apoptosis in IR injury. Furthermore, it prevents damage and restores normal cell function in the kidney following ischemia-reperfusion injury. In renal IR, hsp70 limits apoptosis by controlling the activity of the kinases Akt that regulate the activity of the pro-apoptotic protein Bax. Therefore, the hsp70 levels following renal IR inversely correlate with tubular injury, apoptosis and renal dysfunction^7,8^.

As a circulating hormone in the body, Vitamin D is indispensable for mineral homeostasis and is normally transported in the circulation by the vitamin D binding protein. In the liver, it is hydroxylated on its side chain to form 25-hydroxyvitamin D. However, the kidney is the primary organ where the active form of vitamin D, 1,25-dihydroxyvitamin D3 [1,25(OH)2 D3] or calcitriol is produced. The classical 1,25(OH)2 D3 pathway necessitates the nuclear vitamin D receptor (VDR), which is a transcription factor for 1,25(OH)2 D3 target genes^9^.Vitamin D3 (1, 25(OH)2D3) is the biological active form of vitamin D which exhibits several physiological activities including the regulation of calcium homeostasis, immune functions, anti-fibrotic and antioxidant properties. Furthermore, it reduces cell death and necrosis induced by ROS, produces nephron-protective actions and a candidate intervention role against ischemic invasion might be considered for that. Meanwhile, the active form of vitamin D is produced in the kidney by mitochondria of the proximal tubules. Consequently, vitamin D levels are reduced after renal IR injury^10^.

Erythropoietin (EPO) is a hypoxia-induced hematopoietic hormone, an important protein in production of erythrocytes. It mainly stimulates neovascularisation and angiogenesis, which improves blood flow and is predominantly expressed in the kidney in response to hypoxia. The physiological effects of erythropoietin are mediated by binding to erythropoietin receptors (EPORs), that are found in a variety of locations, including mesangial, glomerular and tubular epithelial cells^11^. The important effects of EPO include reduction in free radicals and proinflammatory cytokines. The recombinant human EPO (rHuEPO) attenuates oxidative stress-associated lipid peroxidation, the influx of inflammatory cell and apoptotic process. It is revealed that EPO can potentially preserve against IR-induced liver, heart and lung injury^12^.

Since vitamin D3 as well as EPO have antioxidant and anti-apoptotic effects and hypoxia/re-oxygenation were shown to contribute to the apoptotic cell death following renal ischemia/reperfusion injury, thus the purpose of this study was to investigate the protective effect of vitamin D and EPO against renal IR damage using the evaluation of hsp70, caspase3 and miR-21 expression.

## Material and methods

### Animals and surgery

For the study, 30 Adult Wistar rats (weighing 200–250 g) were obtained from the experimental animal house. All the protocols and procedures of the study were approved by the Institutional Animal Ethics Committee (ethics code: IR.ARUMS.REC.1399.176). The rats were kept in controlled temperature (21 ± 2 °C) and humidity (60 ± 5%) environment with free access to water ad libitum and food and under 12–12 h light–dark cycle.

For the induction of the anesthesia, 50 mg/kg ketamine and 10 mg/kg xylazine were given by intra-peritoneal injection. The animal was placed at right flank position, after minimal dissection under the last rib, right nephrectomy was performed and the incision was sutured. Then, the rat was placed at left flank position, after minimal dissection under the last rib, left renal pedicle (artery and vein) was exposed. It was occluded by an atraumatic microvascular clamp for 45 min to induce ischemia and then subjected to reperfusion for 24 h. All animals had right nephrectomy and the presence of ischemia was visually confirmed by observing blanching of the kidney.

The rats were randomly divided into five groups of six rats in each group. The control group (N = 6) underwent only nephrectomy without occlusion. The other groups were as follows:

Vehicle + IR group.

Vit D (Vitamin D3) + IR group.

EPO + IR group.

Vit D + EPO + IR group.

Erythropoietin (Neorecormom, Roche, Mannheim, Germany) was administered as a 1000 U/kg single dose IP, 30 min before ischemia^13^. Vitamin D3 used in the present study was obtained from Sigma-Aldrich (USA) and was dissolved in ethanol, diluted with saline and adjusted to a final concentration of 10 mg/kg^14^. Vitamin D3 was injected intraperitoneally, prior to ischemia, an equal amount of saline supplemented with the required volume of ethanol was used as the vehicle.

### Biochemical assays

Blood samples were taken by cardiac puncture, and left kidney tissues were collected after twenty four hours of reperfusion in each group. One part of kidney was snap-frozen in liquid nitrogen for protein and RNA isolation followed by transference to a − 80 °C freezer, and the other part of left kidney was fixed for histological analysis. The blood samples were centrifuged at approximately 2000 rmp for 20 min. The serum blood urea nitrogen (BUN) and creatinine (Cr) levels to assess the renal function were measured, using the Auto-analyser (Alycon 300 USA) with original kits.

### Quantitative real-time polymerase chain reaction (qRT-PCR)

Total RNA was isolated from cells. Quantitative RT-PCR was performed with an ABI 7500 Fast Real Time PCR system (Applied Bio-systems, USA) using a reverse transcriptase kit (Takara) and SYBR Green Master Mix kit (Takara) according to the manufacturer’s instructions. Reverse transcription reaction conditions were processed at 42 °C for 15 min, followed by 3 min at 95 °C. The thermal cycling conditions were processed at 95 °C pre-denaturation, followed by 40 cycles (95 °C for 15 s; 60 °C for 30 s; 72 °C for 60 s). Relative quantification was determined by normalization to U6. The primers are shown in Table 1. All the qRT-PCR expression experiments were performed in triplicates to ensure the reproducibility. The relative gene expression was calculated using 2^−∆∆Ct^ method^15^.

**Table 1:** Sequence of primers used for RT-PCR.

RNA name	Sequence
miR-21 stem	GTCGTATCCAGTGCAGGGTCCGAGGTATTCGCACTGGATACTCAACA
miR-21 forward	CCGCAGGTAGCTTATCAGA
U6 stem	GTCGTATCCAGTGCAGGGTCCGAGGTATTCGCACTGGATACGACAAAAATAT

### Western blot analysis

Proteins were extracted from kidney, and the protein concentrations were measured using a BCA assay kit (TaKaRa BIO INC, Japan). The protein samples were resolved in a 10–12% sodium dodecyl sulfate (SDS)-polyacrylamide gel electrophoresis. Proteins were then transferred to polyvinylidene fluoride (PVDF) membrane, and blocked with 5% nonfat milk in Tris-buffered saline-Tween (TBST) 20 for 2 h at room temperature. Membranes were then incubated with primary antibody overnight. The antibodies were shown as follows: anti-Caspase-3 (1:1000; Santa Cruz Biotechnology), or anti-HSP70 (1:1000; Santa Cruz Biotechnology). An anti-β-actin antibody was used as control. Membranes were washed and incubated for 2 h in the presence of appropriate horseradish peroxidase (HRP)-conjugated secondary antibody. The positive reaction was visualized by using 3, 3′-diaminobenzitine (DAB) solution (Sigma, St. Louis, MO) with a chemiluminescent Immobilon Western blotting detection system^16,17^.

### Histopathological analysis

The renal samples of the experimental animals were sectioned and fixed in 10% buffered-formalin solution and then the sections were embedded in paraffin. Sections of 5 µm were taken, stained with periodic acid Schiff (PAS), and then examined under light microscope in a blinded manner by a pathologist. Histological changes were scored on a 4-point scale: (−) none, (+) mild, (++) moderate, and (+++) severe damage^18^.

### Statistical analysis

All the data were reported as mean ± standard deviation (SD). Significance testing between groups was performed using one-way analysis of variance (ANOVA) followed by multiple comparison procedures using post hoc complementary test, with SPSS version 19.0 and Graph Pad Prism version 8.4.3 (686). A statistical significance was defined at P < 0.05.

## Results

The effect of vitamin D3 and erythropoietin on renal IR injury was investigated in 45 min of renal ischemia followed by 24 h reperfusion. The miR-21 expression is outlined in Fig. 3 and the expression levels of HSP70 and caspase 3 are represented in Figs. 4, 5 and 6. Figure 6 indicates western blot bands for HSP 70 and cleaved caspase-3 protein expression in studying groups and the results of histological evaluation are shown in Table 2.

**Table 2:** Tubular and Glomerular changes in the kidney after 24 h reperfusion (PAS).

Groups	Glomerular atrophy	Acute tubular necrosis	Lymphocyte infiltration	Thickening of basement membrane	Hyaline cast
Control	−	−	−	−	−
IR	+++	+++	+++	+++	+++
Vit D	−	+	++	−	+
EPO	−	+	+	−	++
Vit D + EPO	−	−	+	−	−

A minimum of 10 fields for each kidney slide were examined and assigned for severity of changes using Scores on a scale of: (–) none, (+) mild, (++) moderate, and (+++) severe damage. (n = 6 for each group).

### Effects of ischemia reperfusion

**Figure 1:**
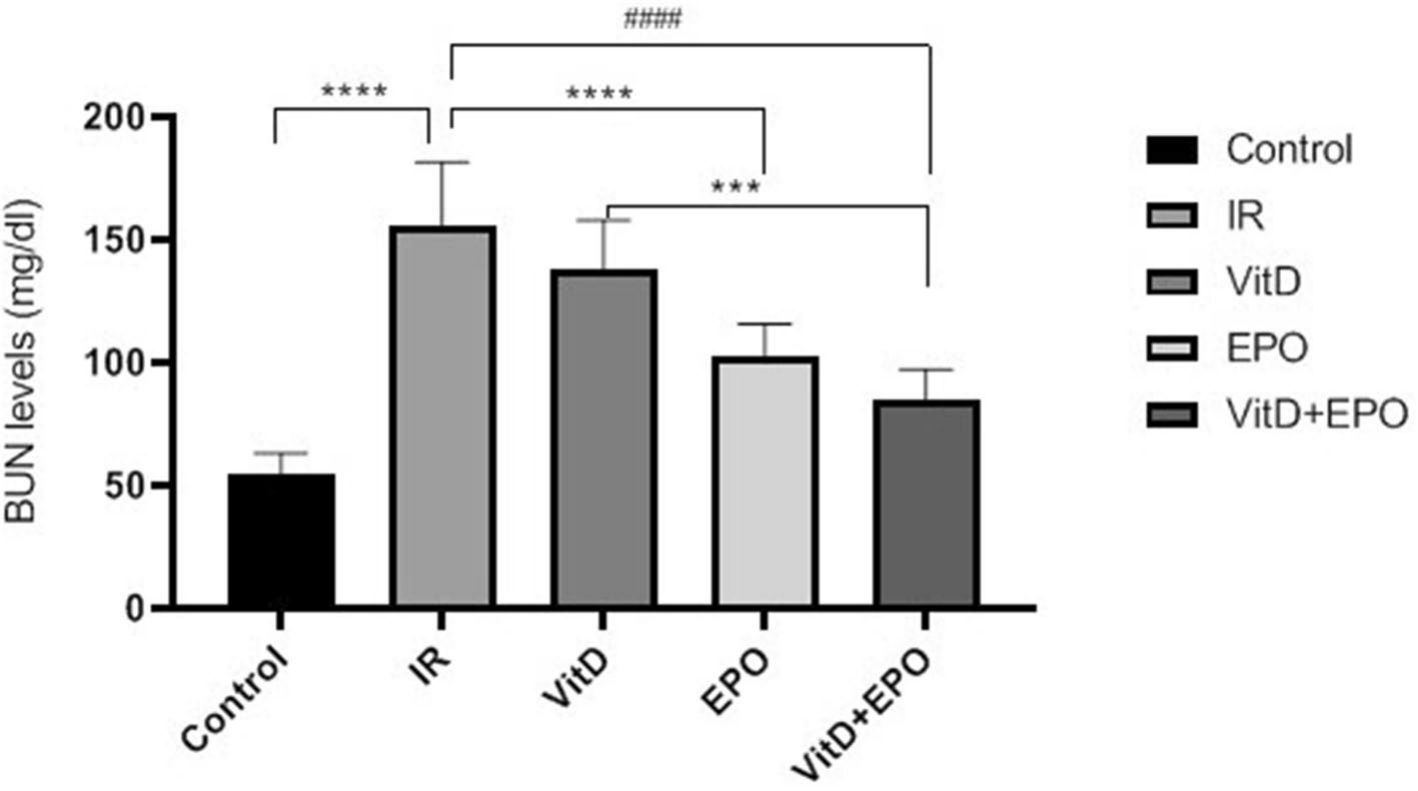
Blood urea nitrogen (BUN) levels. ****P < 0.0001 versus control group. ^**####**^P < 0.0001, ****P < 0.0001 versus IR group. ***P < 0.001 versus Vit D. *IR* Ischemia-reperfusion, *Vit D* vitamin D, *EPO* erythropoietin.

**Figure 2:**
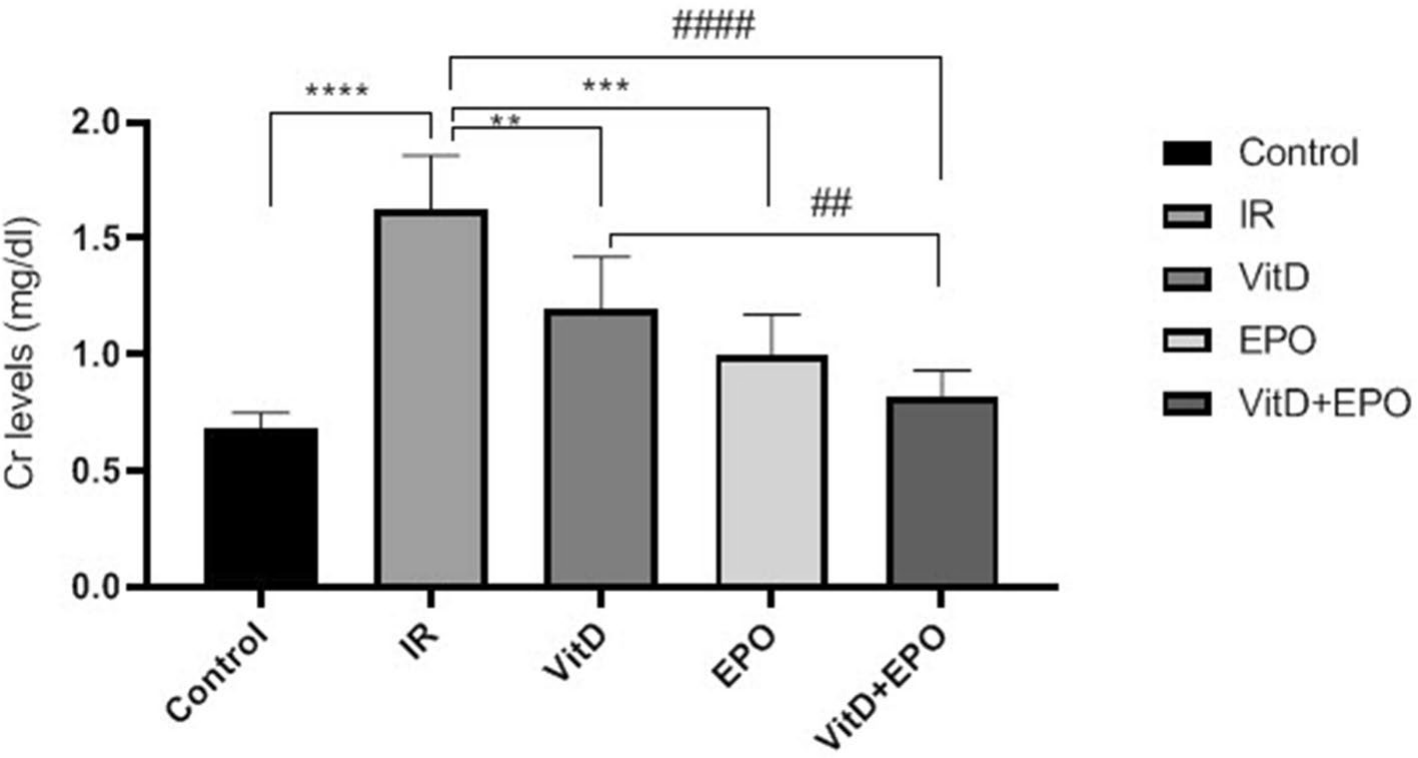
Creatinine (Cr) levels. ****P < 0.0001 versus control group. **P < 0.01, ***P < 0.001, ^**####**^P < 0.0001versus IR group. ^##^P < 0.01 versus Vit D.

**Figure 3:**
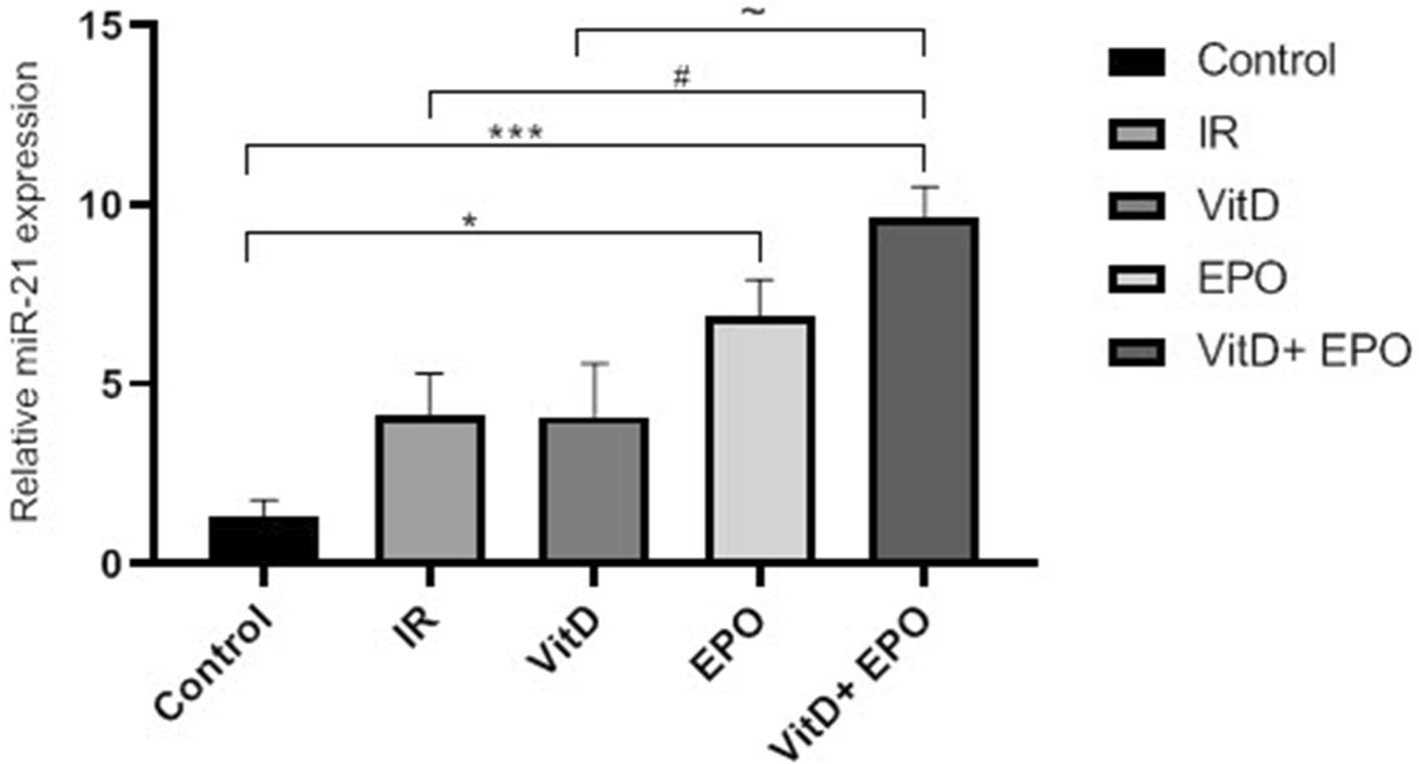
Vitamin D and erythropoietin affect ischemia-reperfusion by regulating miR-21. Relative expression of miR-21 in renal cell was detected by qRT-PCR. Data represent the mean ± SD. *P < 0.05, ***P < 0.001 versus control group. ^**#**^P < 0.05 versus IR group. ^~^P < 0.05 versus Vit D.

**Figure 4:**
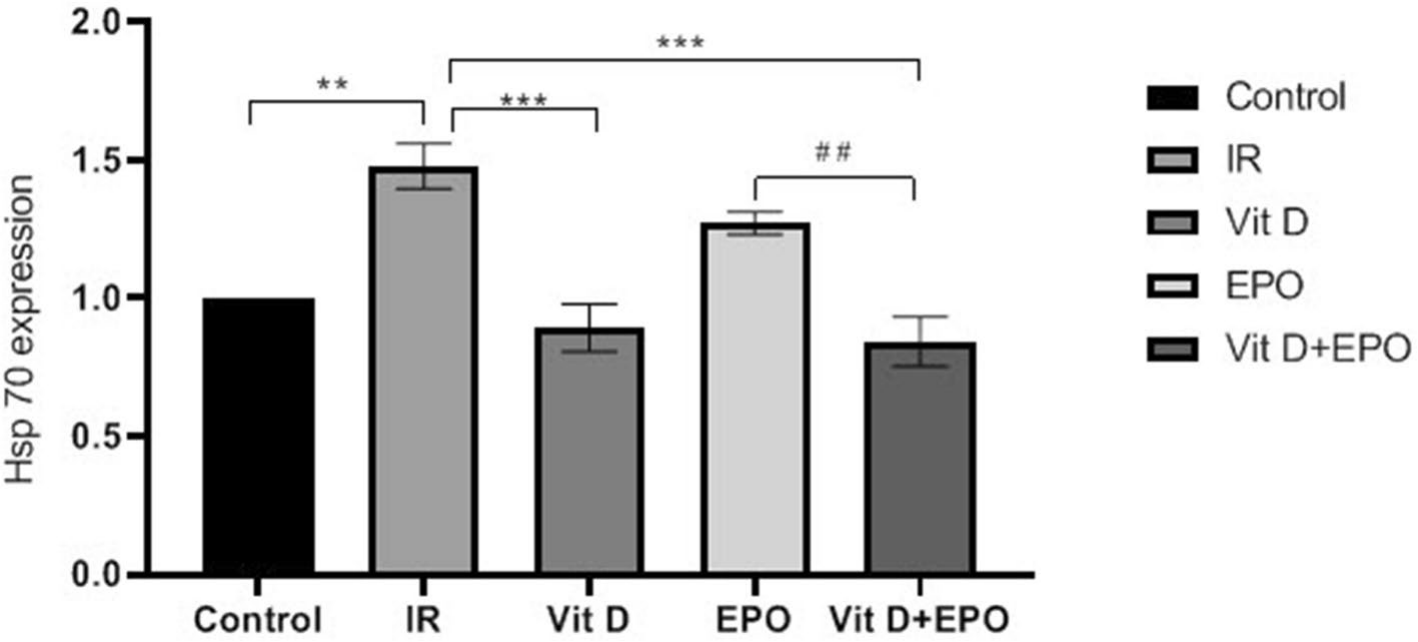
Effect of vitamin D and erythropoietin on ischemia/reperfusion‑induced alterations in HSP 70 protein expression in renal tissue. Protein expression level of HSP70 was detected by western blot analysis. **P < 0.01 versus control group. ***P < 0.001 versus IR group. ^##^P < 0.01 versus EPO group. HSP70; heat shock 70 kDa protein.

**Figure 5:**
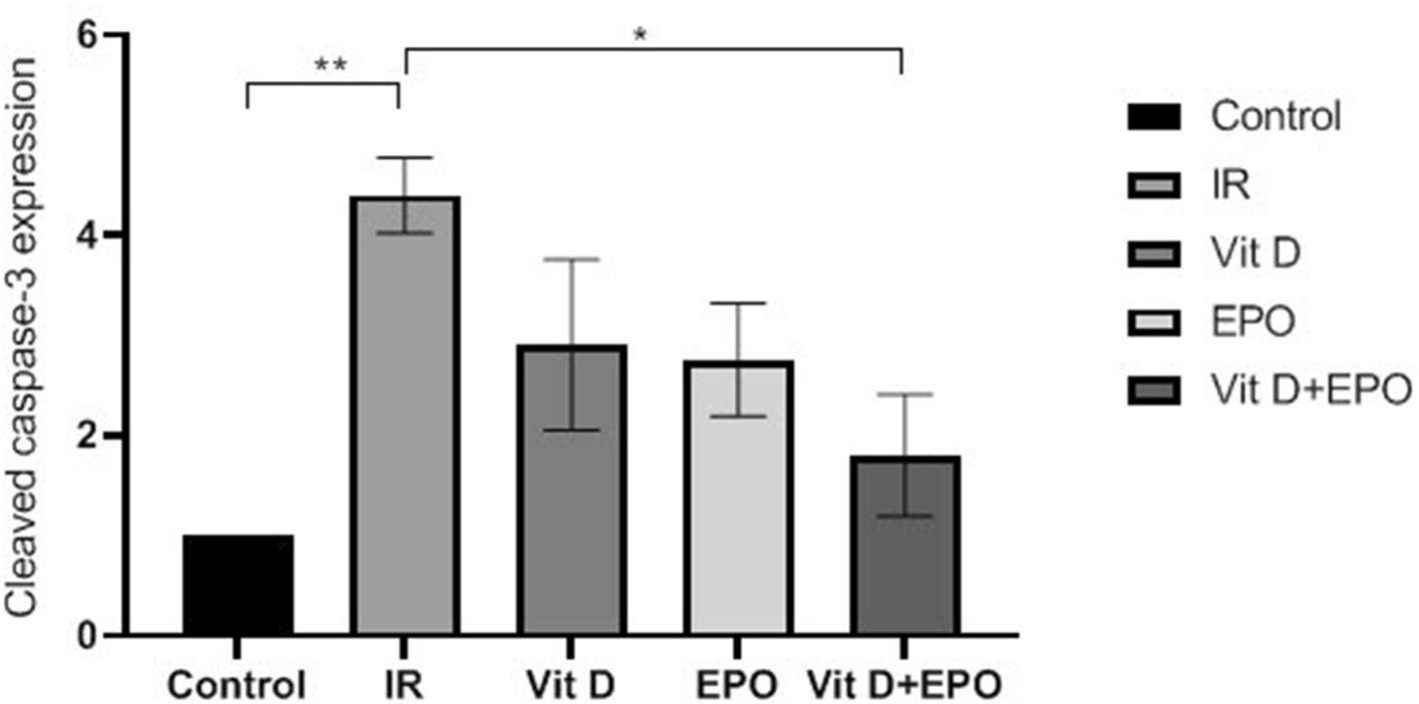
Effect of vitamin D and erythropoietin on the caspase-3 expression after renal IR injury. Cleaved caspase 3 was detected using Western blot. **P < 0.01 versus control group. *P < 0.05 versus IR group.

**Figure 6:**
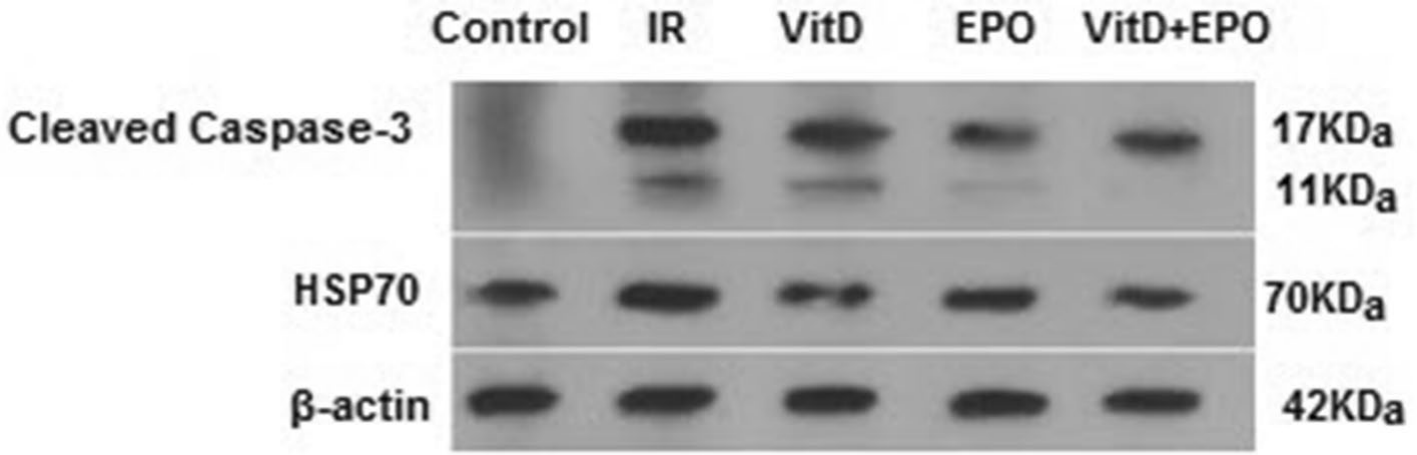
Representative western blot bands for HSP 70 and cleaved caspase-3 protein expression in each group. *IR* Ischemia-reperfusion, *Vit D* vitamin D, *EPO* erythropoietin, *HSP70* heat shock 70 kDa protein.

The BUN-Cr levels in the IR group in comparison to those from control group were significantly higher (P < 0.0001, Figs. 1, 2). An increased expression of MiR-21 in the renal tissues was detected after ischemia-reperfusion. The microarray data were confirmed by qRT-PCR to measure the expression of this miRNA in the samples of the experimental kidney. MiR-21 showed upregulation after ischemia-reperfusion as shown in Fig. 3. The expression of HSP 70 and caspase-3 in IR group was significantly higher as compared with the control group (P < 0.01, Figs. 4, 5). The expression of HSP70 is one of the key mechanisms which works against cellular injury; by inhibiting subsequent caspase activation and decreasing mitochondrial injury, it plays an important role in protecting renal epithelial cells from apoptosis (Fig. 6).

**Figure 7:**
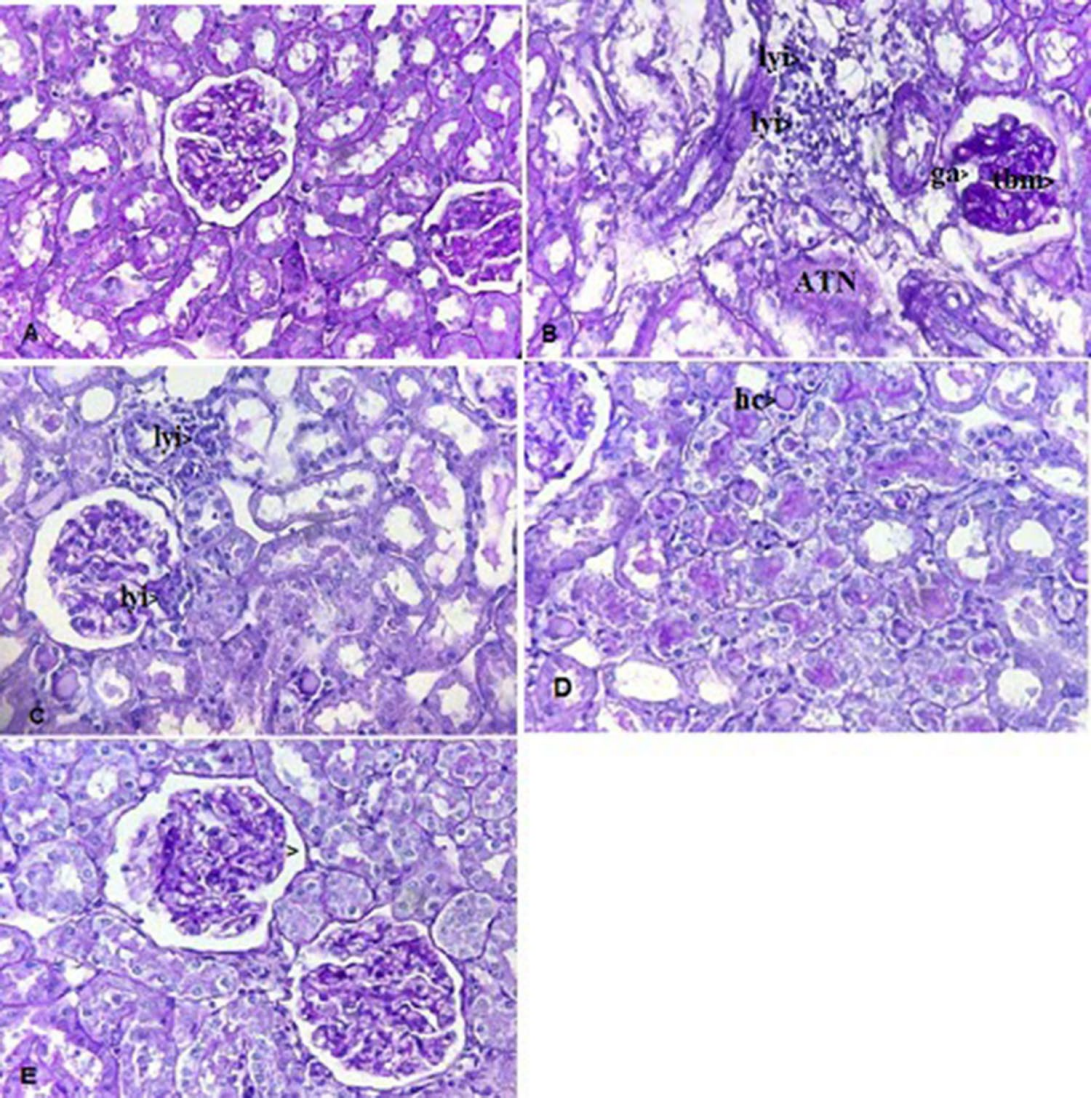
Histopathological evaluation of rat kidneys after ischemia-reperfusion. Kidney sections are stained by periodic acid Schiff (PAS). (**A**) The normal renal tissue structure in the control group. Healthy appearance of glomerular and tubular cells (40 × PAS). (**B**) Thickening of basement membrane (tbm), acute tubular necrosis (ATN), glomerular atrophy (ga), and lymphocyte infiltration (lyi) in IR group. (**C**) The Lymphocyte infiltration (lyi) in the Vit D group. (**D**) The degree of hyaline cast (hc) in the EPO group. (**E**) The normal basement membrane in the Vit D + EPO group (40 × PAS).

Histological assessment of the kidneys showed that there were no morphological changes in the control group (Fig. 7A). In the IR group, thickening of basement membrane, acute tubular necrosis (ATN), glomerular atrophy and lymphocyte infiltration were very obvious (Fig. 7B).

### Effects of vitamin D3 on renal ischemia reperfusion

Serum BUN-Cr levels in the vitamin D3 + IR group were lower than those of the IR group (Figs. 1, 2). The vitamin D-treated rats did not show any changes of miR-21 expression in comparison to the IR group, but this expression was considerably enhanced in comparison to the control group (Fig. 3). HSP 70 and caspase-3 expressions were significantly reduced in the vitamin D group when compared with IR group (P < 0.001, Figs. 4, 5). The administration of vitamin D resulted in noteworthy decline in histopathological changes induced by ischemia reperfusion (Fig. 7C).

### Effects of erythropoietin on renal ischemia reperfusion

Serum BUN-Cr levels in the EPO + IR group were significantly lower than those of the IR group (P < 0.0001, Figs. 1, 2). It was found that in erythropoietin-treated animals, the miR-21 expression was elevated in comparison to the IR group, as erythropoietin significantly upregulated miR-21 when compared with the control (P < 0.05, Fig. 3). In the EPO group, the expression of HSP 70 and caspase-3 were decreased in comparison to IR group (Figs. 4, 5). Also, in the group treated with EPO, there were moderate microstructural alterations in the renal tissues (Fig. 7D).

### Effects of vitamin D3 and erythropoietin on renal ischemia reperfusion

In the vit D + EPO + IR group, the serum level of BUN-Cr was significantly lower than that in the IR group (P < 0.0001, Figs. 1, 2). The study showed that in the group treated with vitamin D and EPO, the miR-21 expression was significantly increased in comparison to the IR group and vitamin D (P < 0.05, Fig. 3). As shown in Figs. 4 and 5, vitamin D and EPO significantly downregulated HSP 70 and caspase-3 expression in comparison to IR group (P < 0.05). Also, vitamin D and EPO co-treatment severely attenuated the histological changes, nearly the normal microstructure of kidney was preserved by vitamin D and EPO pretreatment. Thus, combination therapy appears to have considerable improvement on ischemia–reperfusion injury (Fig. 7E).

## Discussion

Acute kidney injury induced by ischemia-reperfusion injury is a major cause of mortality among patients during partial nephrectomy, especially renal transplantation. Ischemia-reperfusion injury has been reported to result in the development of oxidative stress via the production of free radicals, initiate a series of cellular events that lead to necrotic and apoptotic cell death and increase generation of inflammatory cytokines. Therefore, the investigation and development of effective treatment to reduce renal damage from IR injury is necessary^19,20^.

In this study, renal IR injury resulted in both tubular and glomerular dysfunction. IR significantly enhanced the levels of BUN-Cr, suggesting an impaired glomerular filtration rate which was reduced by vitamin D3 and erythropoietin administration. It is reported that the pretreatment with EPO or vitamin D3 prior to ischemia decreases the renal dysfunction-induced by IR damage and regulates glomerular filtration rate^21,22^. Also, the serum levels of BUN-Cr decreased by the co-administration of two agents (Vit D + EPO), which shows that the co-treatment improves the recovery of renal function.

We found that renal IR elevated the miR-21 expression. Increased expression levels indicate a cellular defensive response to activate apoptotic pathways after renal IR. MiR-21 is an important anti-apoptotic and hematopoietic miRNA^23^. Several studies have suggested that miR-21 expression may be elevated in cells tolerant to hypoxia and may be protective against ischemia-induced hypoxia, so hypoxic conditions are expected to induce the expression of miR-21 in renal epithelial cells^24,25^. The overexpression of miR-21 in renal IR may contribute to the protection of renal epithelial cells against IR injury by attenuating apoptosis of renal tubular cells^26^. Erythropoietin enhanced the expression of miR-21; it was mentioned above that EPO is hematopoietic hormone and miR-21 is a hematopoietic miRNA. Probably this hormone causes the hematopoietic effects through upregulation of miR-21 expression. Also, the co-administration of vitamin D and EPO (Vit D + EPO) significantly increased the miR-21 expression. This finding may indicate that vitamin D and EPO co-treatment decreases the renal IR-induced apoptosis and protects against IR injury by upregulation of miR-21 expression. Furthermore in compatibility with our findings, it was reported that erythropoietin significantly attenuates myocardial IR-induced apoptosis and autophagy activation via up-regulating miR-21 and the protective effect of EPO against myocardial IR injury is associated with regulating miR-21^15^.

Our results demonstrated that IR significantly increased the expression of HSP 70 and caspase-3. Heat shock proteins protect against apoptosis and cell injury. Their expression is upregulated upon exposure to stressful conditions such as hypoxia, oxidative stress and ischemia. It has been indicated that HSP70 protects cells against ischemic injury and accumulates at higher levels after oxidative stress to maintain cell homeostasis^8,27^. In spite of the fact that previous researches have revealed that EPO induced an expression of HSP 70 and resulted in a significant increase in myocardial HSP70 content in the myocardial infarct induced by IR injury^28^, the results of the present study showed that erythropoietin insignificantly decreased the expression of HSP70. Also, the administration of vitamin D especially co-treatment with vitamin D and EPO significantly reduced the hsp70 expression. These findings may show that vitamin D and erythropoietin decreased the magnitude of oxidative stress and ischemic injury, as a result the expression level of hsp70 has been reduced.

Furthermore, we demonstrated that the expression level of caspase-3 was significantly decreased by two agents (VitD + EPO). Caspase-3 is an important apoptosis-related protein within the intrinsic apoptotic pathway. The activity of caspase-3 has been considered as an index of the process of apoptosis^29,30^. Apoptosis is an important cause of renal damage induced by ischemia-reperfusion and IR initiates a series of cellular events that lead to apoptotic cell death. It was revealed that caspase-3 expression is promoted after IR injury, consistent with this finding, we showed that the caspase-3 expression was significantly elevated after IR injury. However, this overexpression of caspase-3 was significantly reduced by the co-administration of vitamin D and EPO. This effect may be due to the following reasons. First, vitamin D and EPO are potent anti-apoptotic agents, which downregulate pro-apoptotic protein (caspase-3) expression^31^. Second, the overexpression of miR-21 by vitamin D and EPO co-treatment could inhibit apoptosis and decrease the expression of caspase-3^32^.

**Figure 8:**
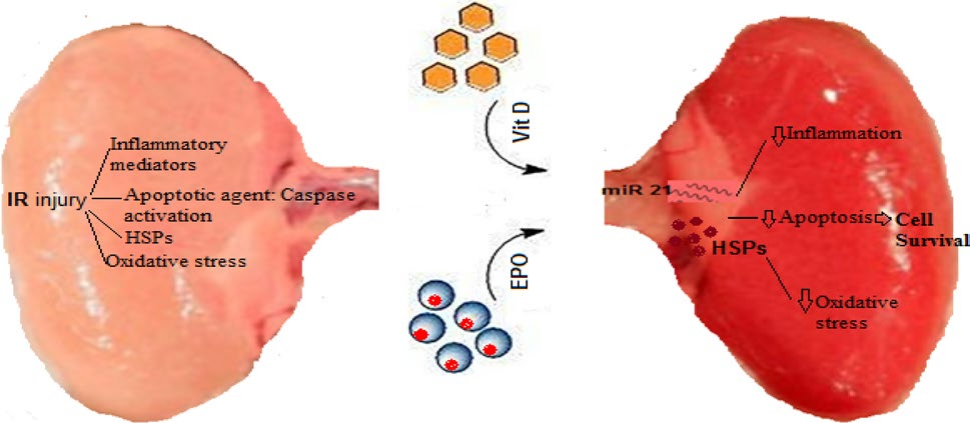
Molecular mechanisms implicated in Vit D/EPO-mediated renoprotection induced by IR injury. *IR* Ischemia-reperfusion, *Vit D* vitamin D, *EPO* erythropoietin, *HSPs* heat shock proteins.

Our histological evaluation showed that ischemia reperfusion caused glomerular and tubular changes as shown by acute tubular necrosis, thickening of basement membrane and lymphocyte infiltration. We found that vitamin D had protective effects on tubular function. Sezgin et al.^21^ reported that vitamin D has protective effects on IR-induced renal injury and the morphological changes are reversed by vitamin D treatment. Erythropoietin treatment decreased the pathological changes associated with renal IR injury. The cyto-protective effects of vitamin D and EPO may be due to their powerful antioxidant and anti-apoptotic properties^31,33^. Also, vitamin D and EPO co-treatment reduced the morphological alterations in renal tissue caused by IR injury, nearly the normal renal structure was preserved by vitamin D + EPO. Therefore, the combination therapy appears to have synergistic protective effects, and to be the most effective compared with the other treatment groups. In a nutshell, Fig. 8 illustrates how the molecular components involved in vitamin D and erythropoietin-mediated renoprotection relate to each other.

### Conclusion

In conclusion, apoptotic cell death and hypoxia are considered to be principal components involved in the pathophysiological tissue changes observed during renal ischemia reperfusion. The administration of vitamin D3 and EPO, which are known antioxidant and anti-apoptotic agents, appears to have protective effects on IR-induced renal injury as indicated by lower expression of caspase-3 and higher expression of miR-21. Also, the co-treatment with vitamin D3 and EPO decreased morphological damage and renal dysfunction. However, the co-administration of vitamin D3 and EPO exerted more nephroprotective effects than single application of them. Therefore, combined therapy may be an alternative option for the decrease of IR-induced renal injury.
